# Waist-to-Height Ratio and Cardiovascular Risk Factors among Chinese Adults in Beijing

**DOI:** 10.1371/journal.pone.0069298

**Published:** 2013-07-12

**Authors:** Li Cai, Aiping Liu, Yumei Zhang, Peiyu Wang

**Affiliations:** 1 Department of Nutrition and Food Hygiene, School of Public Health, Peking University Health Science Center, Beijing, China; 2 Department of Social Medicine and Health Education, School of Public Health, Peking University Health Science Center, Beijing, China; INRA, France

## Abstract

**Objectives:**

To examine whether waist-to-height ratio (WHtR) performed better than, body mass index (BMI) or waist circumference (WC) in relation to hypertension, diabetes, and dyslipidemia among Chinese adults in Beijing.

**Methods:**

A total of 5720 adults (2371 men and 3349 nonpregnant women) aged 18 to 79 years were selected from the general population in a cross-sectional study. Data from a standardized questionnaire, physical examination, and blood sample were obtained.

**Results:**

The area under curve (AUC) values for WHtR (0.661–0.773) were significantly higher than those for BMI for all outcomes in both sexes, except that WHtR and BMI had similar AUCs for dyslipidemia in men. The AUCs for WHtR were significantly higher than those for WC with respect to hypertension in both sexes, and to diabetes in women. AUCs for the relationships between anthropometric indices and the three outcomes were larger in women than in men, and tended to decrease with age. Optimal cutoffs for WHtR were 0.51–0.53 and 0.48–0.50 in men and women, respectively. With regard to the current Chinese criteria for BMI (≥24 kg/m^2^), WC (≥90 cm for men, and ≥85 cm for women), and the recommended cutoff of WHtR (≥0.5), WHtR yielded the greatest odds ratio for hypertension and diabetes in both sexes, and dyslipidemia in women. BMI had the highest odds ratio for dyslipidemia in men. The odds ratios of anthropometric indices for hypertension and diabetes, but not for dyslipidemia, were higher in women than in men. The association between anthropometric indices and the three outcomes decreased with age.

**Conclusion:**

WHtR performed better than BMI and WC for the association with hypertension and diabetes. More studies should be conducted to explore the age differences in the relationships between obesity indices and cardiovascular risk factors.

## Introduction

Cardiovascular disease (CVD) is a major public health problem in the world. It is now well established that obesity substantially increases the risk of hypertension, diabetes, and dyslipidemia, which exerts a great impact on the morbidity and mortality of CVD [1], [2]. Body mass index (BMI) has become the most widely accepted index of overweight and obesity. Much attention worldwide has also been given to the use of waist circumference (WC) for CVD risk assessment and management [3], [4], as WC is strongly correlated with abdominal fat [2]. Recently, the waist-to-height ratio (WHtR) has been proposed as a better screening tool than WC and BMI for adult cardiometabolic risk factors [5]. However, studies remain inconsistent with regard to the use of obesity indices, especially in Asia populations [6]–[9].

Obesity indices of BMI and WC are conventionally used in China. The cutoff of BMI is 24 kg/m^2^, and that of WC is 90 cm for men and 85 cm for women. Although a number of studies have suggested that WHtR of 0.5 is a good cutoff for men and women across many ethnic groups [10], this cutoff has not been used in any clinical guideline in China as the data are often inconclusive. Furthermore, only few studies investigated the discriminatory power of anthropometric indices for dyslipidemia in China, but none of these studies defined dyslipidemia according to the latest Chinese guidelines published in 2007 [11]. The cutoffs for high total cholesterol (TC), high low-density lipoprotein cholesterol (LDL-C), and low high-density lipoprotein cholesterol (HDL-C) were lower in the original guidelines than in the new one. As CVD is the leading cause of mortality in China, it is important to evaluate the practicability and usefulness of WHtR in clinical practice and public health.

In this study, the major outcome variables were hypertension, diabetes, and dylipidemia, which were defined in accordance with the latest guidelines in China. We aimed to: (1) compare the discriminatory power between BMI, WC and WHtR by sex and age; (2) determine the optimal cutoff of WHtR; (3) investigate the strength of association between currently recommended cutoffs for anthropometric indices (i.e., BMI, WC, and WHtR) and CVD risk factors (i.e., hypertension, diabetes, and dyslipidemia) by age and sex.

## Materials and Methods

### Ethics Statement

Ethics approval was obtained from the Ethics Committee of Beijing Centers for Disease Control and Prevention. Written informed consent was acquired from each participant prior to enrollment.

### Study population

In our study, conducted in 2008, the samples were recruited using four-stratified cluster sampling design to represent the general population aged 18 to 79 years in Beijing [12]. After being interviewed at home, participants were invited to attend the physical examination centers at local health stations or community clinics in the participants' residential area where they were examined and provided a blood sample. A total of 6000 people aged 18 to 79 years were invited to participate the study, 5720 of them (2371 men and 3349 nonpregnant women) having complete data for hypertension, diabetes, and dyslipidemia status, sex, age, and the three anthropometric indices (i.e., BMI, WC, WHtR) were included in the analyses.

### Data collection

#### Questionnaire interview

During the study visit, trained interviewers administered a standardized questionnaire to obtain information on demographics and other covariates such as age (year), sex, education attainment (<high school, high school, and college and above), smoking status (never, current, and former), alcohol consumption (never, light, moderate, and at-risk drinker), physical activity (low, moderate and high physical activity), medical history, and family history. Details of covariates were reported elsewhere [12].

#### Measurements

Measurements of standing height, weight, and WC were taken by research staffs trained in accurate measurement techniques. Body height and weight was measured in light indoor clothing without shoes using a standardized protocol. BMI (kg/m^2^) was calculated as weight divided by height squared. WC was measured half way between the lowest rib margin and the iliac crest at the end of a normal expiration. WHtR was calculated as WC (in cm) divided by height (in cm). Two consecutively readings of systolic blood pressure (SBP) and diastolic blood pressure (DBP) were taken on the right arm using a calibrated mercury sphygmomanometer with the participant in a seated position and arm supported at heart level. The participants rested for at least 5 minutes before blood pressure measurement. The mean of these two measures was used in the subsequent analysis. The cuff size was chosen according to the limb circumference.

#### Laboratory assay

Blood samples (12 ml) were collected after 12 hours fast and temporarily conserved in an ice box. The blood samples were centrifuged at 3500×g for 5 minutes at 4°C within 2 hours. All blood samples were refrigerated and analyzed within 5 days. Serum TC, LDL-C, HDL-C, triglycerides (TG) and fasting glucose (FG) were determined using colorimetric methods on a Olympus AU2700 automated biochemistry analyzer (Olympus Optical Co., Tokyo, Japan) by use of commercial reagents (Wako Pure Chemical Industries, Ltd., Osaka, Japan). All procedures were conducted by trained technicians followed standardized protocols.

### Definitions

Hypertension was defined as mean SBP ≥140 mmHg, and/or mean DBP ≥90 mmHg, and/or use of antihypertensive medications in the last 2 weeks. Diabetes was defined as FG level ≥7.0 mmol/L (126 mg/dL), and/or use of pharmacological treatment for diabetes in the last 2 weeks. Dyslipidemia was defined according to the new Chinese criteria [11] as follow: TC ≥6.22 mmol/L (240 mg/dl), and/or LDL-C ≥4.14 mmol/L (160 mg/dl), and/or HDL-C <1.04 mmol/L (40 mg/dl), and/or TG ≥2.26 mmol/L (200 mg/dl), and/or having received treatment for dyslipidemia during the previous 2 weeks.

### Statistical analysis

All the analyses were conducted using SAS software (version 9.1; SAS Institute, Cary, NC). Two-tailed *P*<0.05 was considered to be statistically significant.

The receiver operating characteristic (ROC) curve is a plot of the sensitivity against 1-specificity for each cutoff value. The ROC analysis was performed using BMI, WC, and WHtR as continuous variables in logistic regression models to obtain accurate estimates of area under the curve (AUC) in relation to hypertension, diabetes, and dyslipidemia, respectively. The AUC ranges from 0.5 to 1.0, which is a measure of the discriminative power of a logistic regression model. To facilitate comparisons between WHtR and other indices, SAS macros were used to estimate the AUC and test the difference in the AUC obtained from two logistic regression models [13]. The ROC curve analysis was also used to determine the optimal cutoffs of BMI, WC, and WHtR in relation to the three outcomes. The maximum value of the Youden's index (sensitivity + specificity −1) corresponded to the optimal cutoff of each index for each outcome.

We estimated the odds ratios (ORs) and 95% confidence interval (CI) of the three anthropometric indices in the multiple logistic regression models to estimate the strength of their associations for hypertension, diabetes, and dyslipidemia. In the multiple logistic regression models, body size was graded according to the Chinese specific criteria as follow: (1) BMI ≥24 (yes  = 1, no  = 0) [14]; (2) WC ≥90 cm for men and WC ≥85 cm for women (yes  = 1, no  = 0). In addition, WHtR was categorized into two groups (WHtR ≥0.5; yes  = 1, no  = 0) as recommended [10]. The ORs were adjusted for age, sex, education level, smoking status, drinking, physical exercise, and family history of the corresponding condition.

## Results

Descriptive information of men and women is shown in Table 1. Men had a higher mean BMI, WC, WHtR, SBP, DBP, FG, LDL-C, TG, and a lower mean HDL-C than women. The prevalence of hypertension, diabetes, and dyslipidemia was higher in men than in women.

**Table 1:** Descriptive statistics by sex among adults aged 18–79 years.

	Men (*n* = 2371)	Women (*n* = 3349)
Age (year)	43.4±14.4	44.3±13.6
Height (cm)	170.5±6.4	159.8±5.6
Weight (Kg)	74.0±11.6	62.6±10.0
BMI (kg/m^2^)	25.5±3.7	24.5±3.8
WC (cm)	87.9±8.0	79.6±9.7
WHtR	0.52±0.06	0.50±0.06
SBP (mmHg)	134.1±18.2	126.1±19.2
DBP (mmHg)	83.9±10.7	79.8±10.3
FG (mmol/L)	5.29±1.62	5.06±1.35
TC (mmol/L)	5.06±1.06	5.05±1.01
LDL-C (mmol/L)	3.32±1.05	3.23±0.99
HDL-C (mmol/L)	1.37±0.45	1.59±0.46
TG (mmol/L)	1.80±2.05	1.33±1.19
Hypertension	42.1%	31.0%
Diabetes	9.7%	6.6%
Dyslipidemia	43.3%	30.2%

BMI, body mass index; WC, waist circumference; WHtR, waist-to-height ratio; SBP, systolic blood pressure; DBP, diastolic blood pressure; FG, fasting glucose; TC, total cholesterol; LDL-C, low-density lipoprotein cholesterol; HDL-C, high-density lipoprotein cholesterol; TG, triglycerides.

The ROC curves of the three anthropometric indices in Figure S1 (see Figure S1 for ROC curves of the anthropometric indices for hypertension, diabetes, and dyslipidemia in men and women). Table 2 shows that the AUCs for WHtR were significantly higher than those for BMI with respect to all outcomes in men and women apart from dyslipidemia in men. The AUCs for WHtR were significantly higher than those for WC with respect to hypertension in men and women, to diabetes in women. With regard to dyslipidemia, there was no significant difference in AUCs between WHtR and WC in both men and women. AUCs for the relationships between WHtR and the three outcomes were larger in women than in men, and tended to decrease with age. Similar patterns were observed in the AUCs for BMI, and WC. These patterns were not changed when we excluded those under treatment for the corresponding condition (see Table S1 for Estimates of ROC curve analyses of anthropometric indices for cardiovascular risk factors among those not under treatment for the corresponding condition).

**Table 2:** Estimates of ROC curve analyses of anthropometric indices for cardiovascular risk factors.

	AUC (95%)		
Anthropometric index	Hypertension	Diabetes	Dyslipidemia
All three anthropometric indices by sex			
**Men**			
BMI	0.680 (0.658, 0.701)	0.610 (0.572, 0.648)	0.672 (0.650, 0.693)
WC	0.688 (0.667, 0.710)	0.650 (0.612, 0.687) *	0.668 (0.646, 0.689)
WHtR	0.704 (0.683, 0.725) * †	0.661 (0.625, 0.697) *	0.664 (0.642, 0.685)
**Women**			
BMI	0.735 (0.716, 0.753)	0.698 (0.665, 0.733)	0.679 (0.660, 0.698)
WC	0.765 (0.748, 0.753) *	0.739 (0.707, 0.770) *	0.712 (0.694, 0.730) *
WHtR	0.773 (0.756, 0.790) * †	0.751 (0.720, 0.782) * †	0.714 (0.696, 0.733) *
**BMI by age**			
18–44	0.739 (0.717, 0.762)	0.735 (0.678, 0.792)	0.717 (0.696, 0.738)
45–59	0.677 (0.654, 0.700)	0.617 (0.579, 0.656)	0.609 (0.584, 0.634)
60–79	0.600 (0.556, 0.644)	0.536 (0.483, 0.591)	0.608 (0.568, 0.648)
**WC by age**			
18–44	0.753 (0.731, 0.775)	0.777 (0.728, 0.826)	0.735 (0.715, 0.756)
45–59	0.682 (0.659, 0.705)	0.645 (0.608, 0.682)	0.640 (0.616, 0.664)
60–79	0.600 (0.556, 0.645)	0.558 (0.506, 0.611)	0.592 (0.552, 0.633)
**WHtR by age**			
18–44	0.745 (0.722, 0.767)	0.782 (0.733, 0.832)	0.721 (0.700, 0.742)
45–59	0.683 (0.660, 0.706)	0.643 (0.606, 0.681)	0.625 (0.600, 0.649)
60–79	0.609 (0.565, 0.653)	0.546 (0.494, 0.598)	0.601 (0.560, 0.641)

*P<0.05, Compared to BMI.

† P<0.05, Compared to WC.

ROC, receiver operating characteristics; AUC, area under the curve; BMI, body mass index; WC, waist circumference; WHtR, waist-to-height ratio.

Table 3 shows the optimal cutoffs and their sensitivities and specificities. Optimal cutoffs for BMI were 23.9–25.6 kg/m^2^ in men, and 24.4–25.4 kg/m^2^ in women. Men had a higher optimal cutoff of WC for all the outcomes than women. Optimal cutoffs for WHtR ranged from 0.51 to 0.53 in men, and from 0.48 to 0.50 in women. In men, there was a slight difference among the optimal cutoffs of WHtR in different age groups, while the optimal cutoffs of BMI, and WC varied greatly for different age groups. Women aged 45 years or older had a higher optimal cutoff of BMI, WC, and WHtR for the three outcomes than those aged between 18 and 44 years.

**Table 3:** Optimal cutoffs of anthropometric indices and their sensitivities, specificities, and Youden's indices for cardiovascular risk factors.

		Hypertension				Diabetes				Dyslipidemia			
Anthropometric index													
All three indices by sex		Cutoff	Sen	Spe	YI	Cutoff	Sen	Spe	YI	Cutoff	Sen	Spe	YI
**Men**	BMI	25.6	0.61	0.67	0.28	25.4	0.56	0.59	0.16	23.9	0.81	0.45	0.26
	WC	87.2	0.68	0.61	0.29	88.0	0.64	0.57	0.22	86.0	0.70	0.55	0.26
	WHtR	0.52	0.69	0.64	0.32	0.53	0.50	0.73	0.23	0.51	0.69	0.57	0.26
**Women**	BMI	24.4	0.69	0.67	0.36	25.4	0.62	0.68	0.30	24.4	0.64	0.64	0.29
	WC	80.0	0.69	0.72	0.41	76.0	0.82	0.55	0.37	78.1	0.74	0.60	0.33
	WHtR	0.50	0.72	0.72	0.44	0.48	0.80	0.60	0.39	0.49	0.70	0.64	0.33
**BMI by sex and age**													
**Men**	18–44	25.4	0.68	0.69	0.38	21.3	0.95	0.39	0.34	24.3	0.75	0.60	0.35
	45–59	24.2	0.76	0.46	0.22	25.9	0.50	0.67	0.17	23.9	0.80	0.37	0.17
	60–79	27.1	0.43	0.81	0.24	26.6	0.30	0.76	0.06	26.6	0.46	0.68	0.15
**Women**	18–44	23.8	0.65	0.67	0.33	22.7	0.35	0.87	0.22	23.8	0.63	0.64	0.27
	45–59	25.1	0.68	0.63	0.31	25.4	0.56	0.67	0.23	25.1	0.59	0.56	0.15
	60–79	27.5	0.34	0.86	0.21	24.1	0.49	0.63	0.12	25.6	0.61	0.62	0.23
**WC by sex and age**													
**Men**	18–44	90.0	0.65	0.72	0.37	88.0	0.67	0.68	0.35	87.0	0.64	0.67	0.31
	45–59	87.0	0.67	0.54	0.21	95.0	0.38	0.82	0.21	86.0	0.71	0.47	0.18
	60–79	93.0	0.46	0.74	0.20	93.0	0.33	0.73	0.06	85.0	0.78	0.40	0.18
**Women**	18–44	78.1	0.66	0.72	0.38	76.0	0.46	0.86	0.32	76.0	0.67	0.62	0.29
	45–59	83.0	0.58	0.74	0.31	80.0	0.73	0.56	0.29	80.8	0.60	0.61	0.21
	60–79	87.0	0.53	0.68	0.21	83.5	0.51	0.57	0.08	80.0	0.85	0.34	0.19
**WHtR by sex and age**													
**Men**	18–44	0.51	0.68	0.70	0.38	0.51	0.76	0.70	0.46	0.49	0.75	0.59	0.34
	45–59	0.51	0.69	0.54	0.23	0.56	0.42	0.78	0.20	0.51	0.65	0.49	0.13
	60–79	0.52	0.62	0.55	0.17	0.52	0.54	0.48	0.01	0.52	0.72	0.43	0.15
**Women**	18–44	0.49	0.61	0.73	0.34	0.45	0.42	0.86	0.28	0.46	0.73	0.55	0.29
	45–59	0.50	0.72	0.61	0.33	0.48	0.81	0.45	0.26	0.49	0.74	0.47	0.21
	60–79	0.57	0.37	0.80	0.16	0.53	0.74	0.43	0.16	0.50	0.87	0.34	0.20

Sen, sensitivity; Spe, specificity; YI, Youden's index; BMI, body mass index; WC, waist circumference; WHtR, waist-to-height ratio.

With regard to the current Chinese criteria for BMI (≥24 kg/m^2^), WC (≥90 cm for men, and ≥85 cm for women), and the recommended cutoff of WHtR (≥0.5), sensitivity and specificity were calculated (Table 4). The WHtR cutoff of 0.5 yielded moderate sensitivity and specificity for hypertension and dylipidemia, and largest sensitivity and moderate specificity for diabetes compared with those of BMI, and WC. The WC cutoffs had the lowest sensitivity, and the BMI cutoff had the lowest specificity for all the outcomes in both men and women. The sensitivities of WC, and WHtR cutoffs for hypertension and dyslipidemia, but not for diabetes, increased with age. The specificities of WHtR cutoff decreased from 0.64–0.72 in the 18–44 year group to 0.21–0.28 in the 60 year or older group. BMI and WC cutoffs had similar trends in the association between specificity and age.

**Table 4:** Sensitivities, specificities, and adjusted odds ratios of anthropometric indices for cardiovascular risk factors.

			Hypertension			Diabetes			Dyslipidemia		
Anthropometric index		Cutoff	Sens	Spec	OR (95% CI)*	Sens	Spec	OR (95% CI)*	Sens	Spec	OR (95% CI)*
All three indices by sex											
**Men**	BMI	24	0.79	0.45	2.78 (2.28, 3.39)	0.73	0.36	1.24 (0.90, 1.70)	0.80	0.47	3.31 (2.73, 4.01)
	WC	90	0.61	0.67	2.95 (2.46, 3.54)	0.64	0.58	1.97 (1.46, 2.66)	0.59	0.66	2.59 (2.18, 3.08)
	WHtR	0.5	0.78	0.51	3.14 (2.59, 3.82)	0.79	0.41	2.06 (1.46, 2.90)	0.75	0.50	2.95 (2.45, 3.55)
**Women**	BMI	24	0.75	0.60	3.08 (2.56, 3.70)	0.78	0.51	2.26 (1.60, 3.20)	0.71	0.57	2.49(2.09, 2.95)
	WC	85	0.53	0.84	3.43 (2.85, 4.13)	0.60	0.75	2.40 (1.75, 3.28)	0.46	0.80	2.32 (1.94, 2.77)
	WHtR	0.5	0.74	0.68	3.60 (3.00, 4.33)	0.82	0.58	3.51 (2.38, 5.16)	0.69	0.65	2.81 (2.35, 3.36)
**BMI by age**											
	18–44	24	0.74	0.61	4.05 (3.28, 5.00)	0.84	0.55	5.53 (2.86, 10.7)	0.72	0.63	3.96 (3.27, 4.80)
	45–59	24	0.80	0.43	2.95 (2.39, 3.63)	0.75	0.34	1.47 (1.07, 2.02)	0.76	0.40	2.08 (1.70, 2.54)
	60–79	24	0.75	0.33	1.43 (0.99, 2.05)	0.72	0.27	0.86 (0.57,1.32)	0.80	0.35	2.09 (1.49, 2.93)
**WC by age**											
	18–44	90/85	0.54	0.83	4.75 (3.86, 5.83)	0.63	0.77	4.74 (2.77, 8.09)	0.45	0.83	3.06 (2.52, 3.71)
	45–59	90/85	0.55	0.71	2.83 (2.34, 3.43)	0.60	0.62	2.22 (1.66, 2.95)	0.51	0.68	2.14 (1.77, 2.59)
	60–79	90/85	0.64	0.52	1.83 (1.31, 2.55)	0.65	0.42	1.38 (0.92, 2.07)	0.68	0.50	2.19 (1.61, 2.98)
**WHtR by age**											
	18–44	0.5	0.68	0.71	4.58 (3.74, 5.61)	0.81	0.64	6.54 (3.50, 12.2)	0.62	0.72	3.45 (2.87, 4.16)
	45–59	0.5	0.77	0.49	3.11 (2.54, 3.80)	0.79	0.39	2.19 (1.58, 3.05)	0.74	0.46	2.33 (1.91, 2.84)
	60–79	0.5	0.83	0.28	1.84 (1.24, 2.74)	0.84	0.21	1.57 (0.93, 2.64)	0.88	0.28	2.97 (1.99, 4.42)

*Adjusted odds ratios for cardiovascular risk factors in subjects with versus subjects without high BMI, high WC, or high WHtR, adjusted for age, sex, education level, smoking status, drinking, physical exercise and family history of the corresponding condition.

Sen, sensitivity; Spe, specificity; OR, odds ratios; BMI, body mass index; WC, waist circumference; WHtR, waist-to-height ratio.

The adjusted ORs of anthropometric indices in subjects with versus subjects without high BMI, high WC, or high WHtR are shown in Table 4. The WHtR yielded the greatest OR among the three anthropometric indices for hypertension (OR, 3.14 for men, and 3.60 for women), and diabetes (OR, 2.06 for men, and 3.51 for women) in both men and women, and dyslipidemia (OR, 2.95 for men, and 2.81 for men) in women. The WC had the lowest OR for dyslipidemia, while BMI gave the lowest OR for hypertension, and diabetes in both sexes. In addition, there was no obvious change of the result when cutoff of BMI was defined according to the criterion of World Health Organization as BMI ≥25 Kg/m^2^ (data not shown). The adjusted ORs of anthropometric indices for hypertension and diabetes, but not for dyslipidemia, were higher in women than in men. Results from further analyses conducted by age group indicated that the strongest association between each anthropometric index and each outcome was observed among people aged 18–44 years in the three age groups.

## Discussion

Using population estimates based on a representative sample of Beijing adults, we demonstrated that WHtR was statistically superior to BMI for identifying hypertension, and diabetes in both sexes, and dyslipidemia in women. The WHtR was better than WC in discriminating power for hypertension in both sexes, and diabetes in women. The discriminatory power was larger in women than in men and tended to decrease with age. With regard to the currently recommended cutoff, WHtR had the strongest association with hypertension, and diabetes in both men and women, and dyslipidemia in women, compared to BMI, and WC. These associations were attenuated with age.

The general findings of this study are in agreement with those of previous studies, which showed the superiority of WHtR over BMI in their association with hypertension, and diabetes [15]–[18]. In a recent meta-analysis, Kodama et al. also found WHtR had an association stronger than that of BMI in prediction of diabetes, but concluded that measuring height in addition to WC appeared to have no additional benefit [7]. However, in another meta-analysis, analysis of the within-study difference in AUC showed WHtR to be significantly better than WC for diabetes, hypertension, CVD and all outcomes in men and women [5]. Our findings add evidence to the view that WHtR is better than WC for hypertension, and diabetes. With respect to dyslipidemia, among men there was no significant difference in AUCs between indices, while the WHtR was better than BMI, but similar to WC in discriminating power in women. This is the first study exploring the discriminating power of anthropometric indices for dyslipidemia defined according to the new Chinese guideline. More studies are needed before definitive conclusions can be made.

It has been proposed that a cutoff of WHtR of 0.5 may indicate increased risk for individuals in different ethnic groups [19]. A recent systematic review has provided good evidence to this viewpoint, stating that the same cutoff of WHtR (0·5) can be used for men and women across many ethnic groups [10]. Supporting evidence also comes from studies in China. A prospective study in Tangshan suggested that the cutoff to predict incident diabetes for WHtR was 0.52 and 0.53 in men and women, respectively [20]. A cross-sectional study in Jinan evaluated several CVD risk factors and found that the cutoffs of WHtR were 0.52–0.53 in both sexes [6]. Moreover, using data from the 2002 China National Nutrition and Health Survey, He et al. concluded that a WHtR cutoff of 0.5 for both men and women can be considered as optimum for predicting (pre-) diabetes [21]. Similarly, results from our study showed that the optimal cutoffs of WHtR were 0.51–0.53 in men and 0.48–0.50 in women, respectively.

The optimal cutoffs of WHtR identified in studies from different counties/regions were close but not always equal to 0.5 [5]. However, it is unlikely to apply a country/region-specific cutoff to each population. Thus, logistic regression analyses were performed to estimate the associations between currently recommended cutoff of anthropometric indices and the CVD risk factors. Our results demonstrated that WHtR had the greatest odds ratios for hypertension, and diabetes in both sexes, and dyslipidemia in women.

These analyses supported the suggestion that WHtR may be advantageous because it avoids the need for sex- and ethnic-specific cutoffs and helps to avoid the confusion whereby many different cutoffs for WC have been published for different ethnic groups [19], [22]. Since WHtR is easy and cheap to measure, it is potentially useful in public health. WHtR can be served as a standard screening tool for better comparisons of epidemiological data between different studies.

It is noteworthy that there were age differences in the association of anthropometric indices with hypertension, diabetes, and dyslipidemia in our study. As shown in previous studies, there were age differences in the association of anthropometric indices with hypertension [23], and metabolic risks [24], [25]. In our study, the AUCs for BMI, WC, and WHtR were decreased with age with respective to all outcomes, suggesting weaker discriminating power of anthropometric indices for CVD risk factors in the older group than in the younger group. Furthermore, with regard to the currently recommended cutoff of anthropometric indices, the specificity decreased with age, indicating the false positive rate increased with age. In addition, the association between the anthropometric indices and CVD risk factors was stronger in people aged 18–44 years than those aged 45 years or older. The prevention of obesity to reduce the risk of cardiovascular disease may be more effective in young people. Therefore, more attention should be given to the elimination and prevention of obesity in the young population, although the prevalence of obesity is lower in the young than in the elderly.

These age differences may be partly explained by the fact that the mean value of BMI, WC, and WHtR was increased with age. Moreover, age-associated loss of muscle mass appears inevitable [26]. The older individuals has more central body fat distribution, more intra-abdominal fat than the younger group despite similar BMI values [27]. Taken together, the variations in the deposition of fat mass and lean mass by age may partially explain the differences in the association between anthropometric indices and CVD risk factors as observed in our study. More studies need to be performed to explore the age differences in the relationships between obesity indices and CVD risk factors.

Limitations of our study should be noted. First, the cross-sectional study design precluded establishing causality between the anthropometric indices and the CVD risk factors. Second, false negative results may occur in the classification of diabetes due to a lack of information on 2 h postprandial plasma glucose. Third, cautions may be needed in the interpretation of the optimal cutoffs of anthropometric indices in certain subgroups.

In conclusion, with respective to the currently recommended cutoffs, WHtR may be superior to BMI, and WC in the association with prevalent hypertension, and diabetes in both sexes, despite that WHtR and WC had similar discriminating power for diabetes in men. Both the discriminating power of anthropometric indices and their association with hypertension, diabetes, and dyslipidemia tended to decrease with age in men and women, while the optimal cutoffs appeared to increase with age in women. Future studies evaluating the association between anthropometric indices and CVD risk factors should take age into account.

## Supporting Information

Figure S1 **ROC curves of the anthropometric indices for hypertension, diabetes, and dyslipidemia in men and women.**
(DOC)

Table S1 **Estimates of ROC curve analyses of anthropometric indices for cardiovascular risk factors among those not under treatment for the corresponding condition.**
(DOC)
