# Supplementary material for: Waist-to-Height Ratio and Cardiovascular Risk Factors among Chinese Adults in Beijing
Source: PLoS One. 2013 Jul 12;8(7):e69298. doi: 10.1371/journal.pone.0069298 (PMC3709905; doi:10.1371/journal.pone.0069298)
Supplement: Figure S1 — ROC curves of the anthropometric indices for hypertension, diabetes, and dyslipidemia in men and women. (DOC) [file pone.0069298.s001.doc]

Figure S1. ROC curves of the anthropometric indices for hypertension, diabetes, and dyslipidemia in men and women


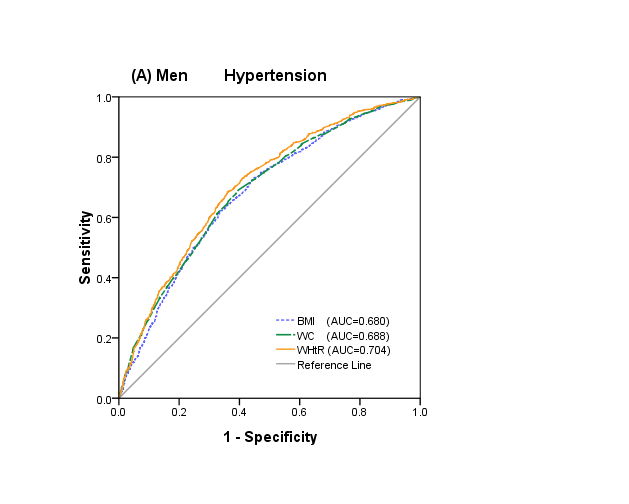

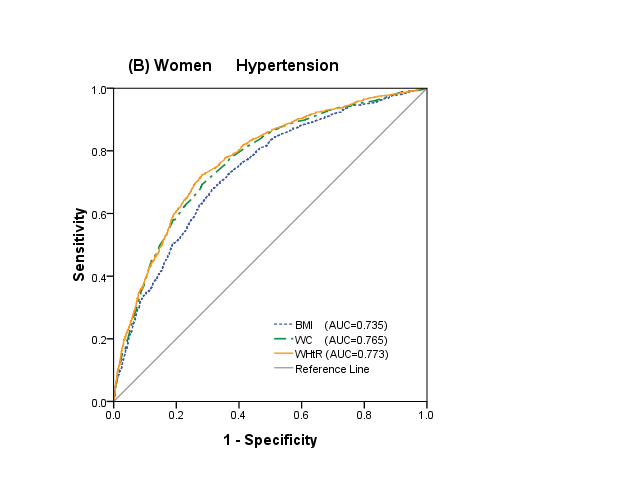


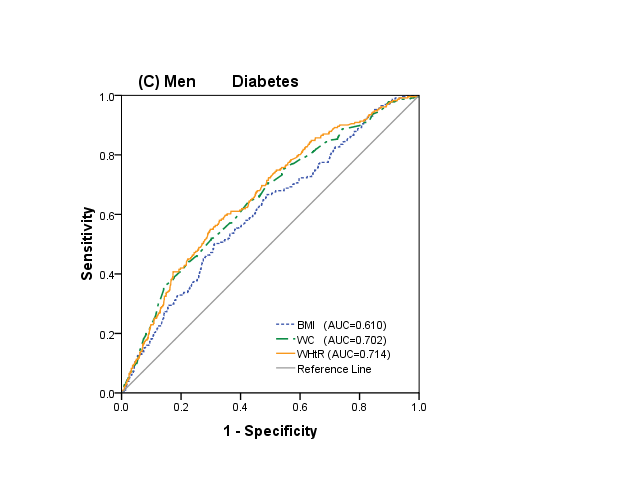

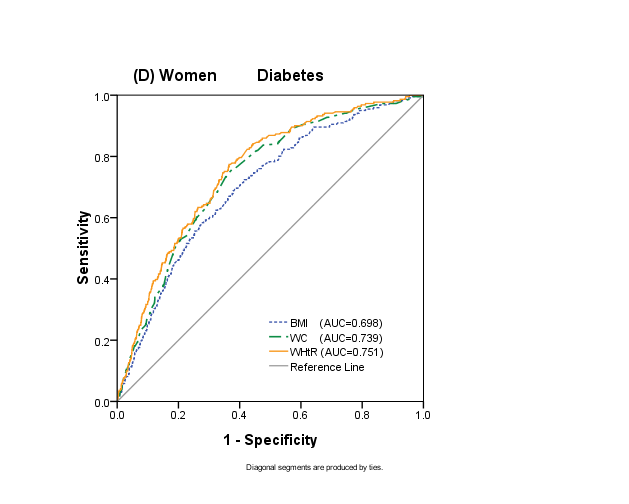


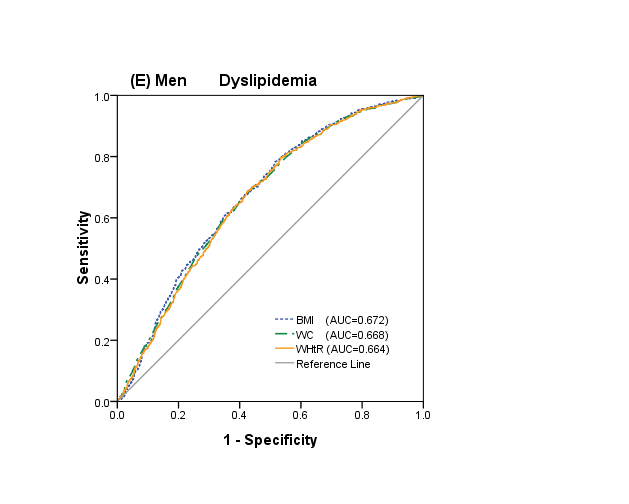

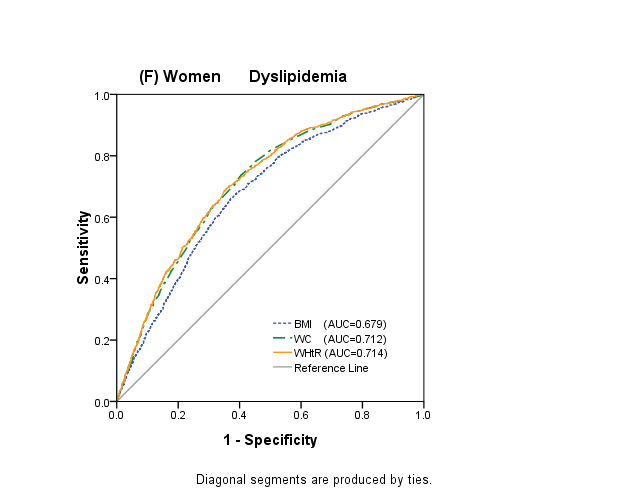


AUC, area under the curve; BMI, body mass index; WC, waist circumference; WHtR, waist-to-height ratio.
