# Supplementary material for: Waist-to-Height Ratio and Cardiovascular Risk Factors among Chinese Adults in Beijing
Source: PLoS One. 2013 Jul 12;8(7):e69298. doi: 10.1371/journal.pone.0069298 (PMC3709905; doi:10.1371/journal.pone.0069298)
Supplement: Table S1 — Estimates of ROC curve analyses of anthropometric indices for cardiovascular risk factors among those not under treatment for the corresponding condition. (DOC) [file pone.0069298.s002.doc]

Table S1 Estimates of ROC curve analyses of anthropometric indices for cardiovascular risk factors among those not under treatment for the corresponding condition

|  | **AUC (95%)** | | |
| --- | --- | --- | --- |
| **Anthropometric index** | **Hypertension ^a^** | **Diabetes ^b^** | **Dyslipidemia ^c^** |
| **All three anthropometric indices by sex** | |  |  |
| **Men** | |  |  |
| BMI | 0.671 (0.647, 0.695) | 0.619 (0.569, 0.669) | 0.671 (0.649, 0.694) |
| WC | 0.671 (0.646, 0.695) | 0.655 (0.604, 0.705) | 0.664 (0.642, 0.686) |
| WHtR | 0.684 (0.661, 0.708)^†^ | 0.667 (0.619, 0.716) | 0.660 (0.638, 0.682) |
| **Women** |  |  |  |
| BMI | 0.736 (0.714, 0.758) | 0.743 (0.700, 0.786) | 0.679 (0.658, 0.699) |
| WC | 0.759 (0.738, 0.781)^*^ | 0.759 (0.720, 0.798) | 0.711 (0.692, 0.730)^*^ |
| WHtR | 0.761 (0.740, 0.782)^*^ | 0.766 (0.726, 0.805) | 0.711 (0.692, 0.731)^*^ |
| **BMI by age** |  |  |  |
| 18-44 | 0.740 (0.715, 0.764) | 0.754 (0.686, 0.821) | 0.717 (0.696, 0.738) |
| 45-59 | 0.678 (0.651, 0.705) | 0.642 (0.593, 0.692) | 0.607 (0.581, 0.632) |
| 60-79 | 0.580 (0.528, 0.631) | 0.572 (0.495, 0.649) | 0.617 (0.575, 0.658) |
| **WC by age** |  |  |  |
| 18-44 | 0.753 (0.729, 0.777) | 0.804 (0.750, 0.857) | 0.736 (0.715, 0.757) |
| 45-59 | 0.689 (0.663, 0.716) | 0.662 (0.616, 0.708) | 0.639 (0.614, 0.664) |
| 60-79 | 0.583 (0.531, 0.634) | 0.546 (0.466, 0.625) | 0.594 (0.552, 0.636) |
| **WHtR by age** |  |  |  |
| 18-44 | 0.739 (0.714, 0.764) | 0.816 (0.763, 0.869) | 0.721 (0.700, 0.742) |
| 45-59 | 0.688 (0.661, 0.714) | 0.660 (0.612, 0.709) | 0.622 (0.597, 0.647) |
| 60-79 | 0.578 (0.526, 0.629) | 0.546 (0.469, 0.624) | 0.606 (0.564, 0.648) |

^*^ P<0.05, Compared to BMI.

^†^ P<0.05, Compared to WC.

^a^ Those under treatment for hypertension were excluded (n=4986).

^b^ Those under treatment for diabetes were excluded (n=5492).

^c^ Those under treatment for dyslipidemia were excluded (n=5501).

ROC, receiver operating characteristics; AUC, area under the curve; BMI, body mass index; WC, waist circumference; WHtR, waist-to-height ratio.
